# Facial profile esthetics in operated children with bilateral cleft lip and palate

**DOI:** 10.1590/2177-6709.22.4.041-046.oar

**Published:** 2017

**Authors:** Rita de Cássia Moura Carvalho Lauris, Leopoldino Capelozza, Louise Resti Calil, José Roberto Pereira Lauris, Guilherme Janson, Daniela Gamba Garib

**Affiliations:** 1 Universidade de São Paulo, Hospital de Reabilitação de Anomalias Craniofaciais, Departamento de Ortodontia (Bauru/SP, Brasil).; 2 Universidade do Sagrado Coração, Programa de Pós-graduação em Ortodontia, Departamento de Biologia Oral (Bauru/SP, Brasil).; 3 Universidade de São Paulo, Faculdade de Odontologia de Bauru, Departamento de Ortodontia (Bauru/SP, Brasil).

**Keywords:** Cleft lip, Cleft palate, Esthetics, Rehabilitation.

## Abstract

**Objective:**

The aim of this study was to evaluate the facial profile esthetics of rehabilitated children with complete bilateral cleft lip and palate (BCLP), comparing the judgment of professionals related and not related to cleft rehabilitation and laypersons.

**Methods:**

Thirty children in the mixed dentition (24 male; 6 female) with a mean age of 7.8 years were evaluated using facial profile photographs by 25 examiners: 5 orthodontists and 5 plastic surgeons with experience in cleft care, 5 orthodontists and 5 plastic surgeons without experience in oral cleft rehabilitation and 5 graduated laymen. Their facial profiles were classified into esthetically unpleasant (grade 1 to 3), esthetically acceptable (grade 4 to 6), and esthetically pleasant (grade 7 to 9). Intraexaminer and interexaminer errors were evaluated using Spearman correlation coefficient and Kendall’s test, respectively. Inter-rater differences were analyzed using Friedman test and Student-Newman-Keuls test for multiple comparisons.

**Results:**

Orthodontists dealing with oral clefts rehabilitation considered the majority of the sample as esthetically pleasant. Plastic surgeons of the cleft team and laypersons classified most of the sample as esthetically acceptable. Most of the orthodontists and plastic surgeons not related to cleft care evaluated the facial profile as esthetically unpleasant. The structures associated to unpleasant profiles were the nose, the midface and the upper lip.

**Conclusions:**

The facial profile of children with BCLP was classified as esthetically acceptable by laypersons. Professionals related to cleft rehabilitation were more lenient and those not related to cleft care were stricter to facial esthetics than laypersons.

## RESUMO

**Objetivo:**

o objetivo desse estudo foi avaliar a estética do perfil facial de crianças com fissura labiopalatina completa bilateral (FLCB) operadas, comparando o julgamento de leigos e de profissionais relacionados e não relacionados à reabilitação de fissuras.

**Métodos:**

fotografias do perfil facial de trinta crianças na dentadura mista (24 do sexo masculino e 6 do sexo feminino), com idade média de 7,8 anos, foram avaliadas por 25 examinadores: 5 ortodontistas e 5 cirurgiões plásticos com experiência em fissuras, 5 ortodontistas e 5 cirurgiões plásticos sem experiência na reabilitação de fissuras, e 5 leigos com formação superior. Os perfis faciais foram classificados como esteticamente desagradável (grau 1 a 3), esteticamente aceitável (grau 4 a 6) e esteticamente agradável (grau 7 a 9). Para verificar a concordância intraexaminadores e interexaminadores, foram utilizados o Coeficiente de Correlação de Spearman e o Coeficiente de Concordância de Kendall, respectivamente. Para comparação entre as categorias de acordo com os escores atribuídos, foram aplicados o teste de Friedman e o teste de Student-Newman-Keuls, para comparações múltiplas.

**Resultados:**

os ortodontistas que lidam com reabilitação de fissuras labiopalatinas consideraram a maioria da amostra como esteticamente agradável. Cirurgiões plásticos com experiência em fissuras e leigos classificaram a maioria da amostra como esteticamente aceitável. Grande parte dos ortodontistas e cirurgiões plásticos sem experiência na reabilitação de fissuras classificou o perfil facial como esteticamente desagradável. As estruturas associadas aos perfis desagradáveis foram o nariz, o terço médio da face e o lábio superior.

**Conclusões:**

o perfil facial de crianças com FLCB foi classificado como esteticamente aceitável pelos leigos; já os profissionais relacionados à reabilitação de fissuras foram mais complacentes, em comparação aos profissionais sem experiência na reabilitação de fissuras, que, por sua vez, foram mais rigorosos quanto à estética facial do que os leigos.

## INTRODUCTION

Complete bilateral cleft lip and palate (BCLP) is the most severe manifestation of oral clefts, corresponding to 14% of all types of oral clefts.^1^ BCLP causes serious esthetical, functional, anatomic and psychosocial disorders requiring early surgery repair.^2^ The rehabilitation protocol for BCLP includes lip and palate repair, alveolar bone graft and orthodontics, isolated or combined with orthognatic surgery.^3^ At birth, a patient with complete BCLP presents severe convex facial profile due to premaxilla projection.^4^ After lip repair, protrusion of the premaxilla decreases and maxillary deficiency may be observed during growth.^5^ According to Semb,^5^ in patients with BCLP, the maxilla is prominent at 5 years of age. At 7 years of age the maxillary prominence is similar to individuals without cleft. At 18 years of age, the maxilla shows severe retrusion. Maxillary growth deficiency decreases facial convexity in BCLP. Additionally, patients with BCLP show hyperdivergent growth of the mandible determining a posteriorly positioned chin.^3^

In patients with BCLP, besides the maxillary deficiency, the upper lip scar, columella length and nasal morphology may impair facial esthetics.^6^ ^-^ ^9^ In the few studies that judged facial esthetics of patients with oral clefts, there is consensus regarding the dissatisfaction with the cosmetic results obtained by the professionals involved in their rehabilitation, as well as by the patients themselves.^10^ ^,^ ^11^ Chetpakdeechit et al^12^ analyzed the facial esthetic outcome of patients with BCLP after orthodontic treatment and found that the upper lip, the nose and the scar were negative features affecting the esthetical evaluation. A recent study has evaluated the facial profile esthetics of BCLP after complete rehabilitation and classified most of the sample as esthetically acceptable.^13^

Evaluation of facial esthetics is extremely important in order to study the outcome of treatment protocols.^1^ The main goals of rehabilitation is reaching good facial esthetics and speech intelligibility.^11^ The few previous studies on facial esthetics evaluation in CLP were performed in adulthood after complete rehabilitation.^11^ ^-^ ^16^ However, facial appearance in the school age is very important for children interrelationship, sociability, self-esteem and learning productivity.^17^ ^-^ ^19^ Therefore, the aim of this study was to evaluate the esthetics of facial profile in children with complete BCLP and compare the assessment of laypersons and professionals related and not related to cleft rehabilitation.

## MATERIAL AND METHODS

This cross-sectional study was approved by the Ethical Committee of our institutional review board (protocol number 438/2002) and an informed consent was obtained. The study sample comprised 30 children in the mixed dentition with complete BCLP consecutively selected during the year of 2004 at a single center. The selection was performed during the first orthodontic appointment. The inclusion criteria were: Mediterranean descents and absence of syndromes. The sample included 24 males and 6 females with a mean age of 7.8 years of age (ranging from 5.6 to 10.3 years).

All patients were operated by a plastic surgeon of *Hospital de Reabilitação de Anomalias Craniofaciais* team following the current protocol of the hospital which includes one-stage lip repair with Spina technique at 3 to 6 months of age and palate repair with Von Langenback technique at 12 months of age. No pre-surgical orthopedics was used. In most cases, nasal columella elongation was performed at 6.3 years of age on average. The evaluation was performed before secondary bone graft procedure.

The photographs were taken by the same examiner using a natural head position.^20^ ^,^ ^21^ Patients were instructed to have the teeth occluded and the lips relaxed.

Right and left facial profile photographs were taken from each patient. The obtained images were transferred to a computer and printed in a 10 x 15 cm size. The photographs were evaluated by twenty-five examiners divided into five groups: 5 orthodontists with experience in rehabilitation of oral clefts (ODC), 5 orthodontists with no experience in cleft treatment (ONC), 5 plastic surgeons with experience in oral clefts (PSDC), 5 plastic surgeons with no experience in cleft treatment (PSNC) and 5 laypersons (1 veterinarian, 1 engineer, 2 lawyers and 1 agronomist). All the professionals with experience in oral clefts worked at the center where the study was conducted.

Each examiner received an album with the sample of 60 photographs. Both facial profiles of each patient were positioned in the same page for simultaneous visualization. No identification of the presence of cleft was provided. The raters were instructed to perform the assessment within approximately 30 seconds for each photograph, assigning scores from 1 to 9 according to Ferrari Jr. et al.^13^

**Figure 1:**
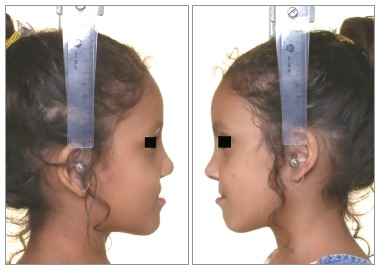
Patient who obtained the lowest score (2.8).

**Figure 2:**
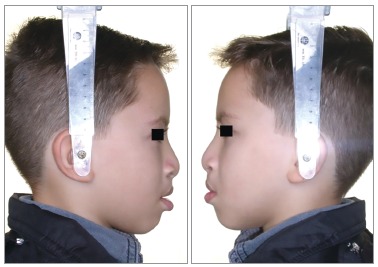
Patient who obtained an intermediate score (4.4).

**Figure 3:**
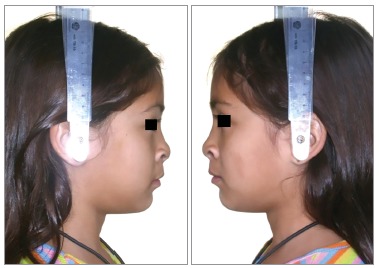
Patient who obtained the highest score (6.2).

Facial profile was considered esthetically unpleasant for scores 1 to 3; esthetically acceptable for scores 4 to 6, and esthetically pleasant for scores 7 to 9 (Figs 1 to 3, respectively). When the score assigned was 1 to 3, the examiner was requested to identify the facial structures responsible for the unpleasant aspect. The photographs were evaluated twice by the 25 examiners with an interval of 30 days between both evaluations.

### Method error

Intraexaminer errors were evaluated using Spearman correlation coefficient. Interexaminer error was evaluated using Kendall Coefficient of Agreement (W).

### Statistical analyses

Inter-rater differences were compared using Friedman test and Student-Newman-Keuls test for multiple comparisons. The significance level regarded was 5%.

## RESULTS

Intraexaminer agreement was statistically significant for all the rater categories and the coefficient of agreement varied from 0.46 to 0.68 (Table 1). There was statistically significant interexaminer agreement within all categories of raters and the coefficient of agreement varied from 0.55 to 0.74 (Table 2).

**Table 1:** Intraexaminer agreement for each category of raters (Spearman correlation coefficient).

Category	r_S_	p
ODC	0.63	0.0001*
ONC	0.68	0.0001*
PSDC	0.56	0.0001*
PSNC	0.46	0.0001*
L	0.51	0.0001*

*Statistically significant at *p* < 0.05.

ODC: orthodontists dealing with cleft; ONC: orthodontists with no experience in cleft; PSDC: plastic surgeons dealing with cleft; PSNC: plastic surgeons with no experience in cleft; L: laymen.

**Table 2:** Interexaminer agreement for each category of raters (Kendall’s coefficient of agreement).

Category	W	p
ODC	0.74	0.0001*
ONC	0.64	0.0001*
PSDC	0.57	0.0001*
PSNC	0.64	0.0001*
L	0.55	0.0001*

*Statistically significant at *p* < 0.05.

ODC: orthodontists dealing with cleft; ONC: orthodontists with no experience in cleft; PSDC: plastic surgeons dealing with cleft; PSNC: plastic surgeons with no experience in cleft; L: laymen.

There were significant differences among all rater categories except between ONC and PSNC. The ODC and PSDC assigned the highest scores for facial esthetics compared to the other raters (Table 3). ONC and PSNC assigned the lowest scores. Laymen gave intermediate scores between professionals related and non related to cleft care.

**Table 3:** Interexaminer comparisons for scores of facial profile esthetics (Friedman and Student-Newman-Keuls tests).

ODC	ONC	PSDC	PSNC	L	p
Mean (SD)	Mean (SD)	Mean (SD)	Mean (SD)	Mean (SD)	
6.4 (0.61)^a^	3.2 (0.74)^b^	5.1 (0.99)^c^	3.2 (0.26)^b^	4.0 (1.26)^d^	0.0001

Different letters show statistically significant differences (Student-Newman-Keuls test).

ODC: orthodontists dealing with cleft; ONC: orthodontists with no experience in cleft; PSDC: plastic surgeons dealing with cleft; PSNC: plastic surgeons with no experience in cleft; L: laymen.

The frequency of “esthetically unpleasant” scores was very low for ODC and PSDC (Table 4). In contrast, ONC and PSNC classified more than 50% of the sample as having unpleasant profiles.

**Table 4:** Percentage of patients classified as unpleasant by different category of raters.

Category	%
ODC	3.30
ONC	63.3
PSDC	6.70
PSNC	70.0
L	40.0

ODC: orthodontists dealing with cleft; ONC: orthodontists with no experience in cleft; PSDC: plastic surgeons dealing with cleft; PSNC: plastic surgeons with no experience in cleft; L: laymen.

**Figure 4:**
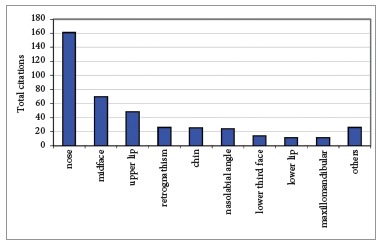
The structures associated to the worst scores were the nose, the midface and the upper lip.

The structures most frequently pointed as responsible for the unpleasant profile were the nose and the midface (Fig 4).

## DISCUSSION

The concept of beauty is very subjective and varies individually.^11^ Despite the subjectivity of beauty interpretation, there was good intra and interexaminer agreement.^11^ ^,^ ^13^ Previous studies also showed good reproducibility of the subjective facial analysis method.^11^ ^,^ ^13^ Additionally, splitting the raters by category may have decreased the variation between them.

Differences for the evaluation of facial esthetics were observed for the different types of raters (Table 3). Orthodontists and plastic surgeons dealing with oral clefts (ODC and PSDC) scored most of the patients as esthetically acceptable even though the mean score was lower for the plastic surgeons. On the other hand, orthodontists and plastic surgeons not related to cleft care (ONC and PSNC) classified the facial profile of the sample as esthetically unpleasant. Laymen attributed an intermediate score between professionals related and not related to cleft care, considering the majority of the sample as esthetically acceptable. From the social point of view, layperson evaluation is more important because they represent the way society see the patient. The esthetics that pleases the patient’s fellows has great significance in building self-esteem and inter-personal relationship.^22^ The layperson opinion is also very important considering the high frequency of bullying among patients with oral clefts.^23^ ^,^ ^24^

Why did professionals related to cleft care classified the sample with a much better score compared to laypersons? There was also a discrepancy between the frequency of esthetically unpleasant classification between professionals related to cleft care and laypersons (Table 4). While ODC and PSDC classified less than 10% of the sample as esthetically unpleasant, laymen scored 40% of the patients with the worse scores. Previous studies also verified that professionals related to cleft care were more lenient with facial esthetics at the end of the rehabilitation process.^11^ ^,^ ^13^ According to these studies, professionals dealing with cleft rehabilitation recognize the limitations of treatment and are more tolerant with morphologic deviations.^7^ ^,^ ^11^ ^,^ ^13^ Only one study found similarity in esthetic evaluation between plastic surgeons related to cleft care and laypersons, however the study considered only the nasal esthetic outcome of BCLP after secondary nasal reconstruction.^25^ Both raters considered the nose with acceptable esthetics even though far from the ideal.^25^

Professionals not constantly dealing with oral cleft rehabilitation have normality and perfection as comparative parameters and therefore are more strict in their evaluation.^11^ For this reason, they considered most of the profiles as esthetically unpleasant (Table 4). This explains the deficient results compared to normality, which is also an important information for cleft care professionals. 

Regarding the structures recognized as responsible for the lowest scores, the nose was the most cited for all categories of raters (Fig 4). The nose is largely affected by bilateral cleft lip and palate demonstrating a very short columella and a flat nose ala at birth.^4^ These results show the need for nose surgery in these patients, which is frequently performed at the end of the rehabilitation process, after growth.^4^ It is speculated that early plastic surgery of the nose could further impair maxillary growth and therefore it is postponed to the end of the rehabilitation process. The second and third structures most often cited as responsible for unpleasant profiles was the deficient midface and the retruded upper lip (Fig 4). Lip and palate repair cause progressive maxillary retrusion during growth,^5^ resulting in retruded midface and upper lip. These results show that maxillary growth deficiency can influence facial esthetics beginning at an early age. In summary, the structures that impair facial esthetic evaluation are those most affected by the cleft itself or by the primary interventions.

## CONCLUSIONS

» The facial profile of rehabilitated children with complete BCLP was most frequently scored as esthetically acceptable.
» Professionals related to oral cleft rehabilitation gave better scores for facial esthetics than laypersons and professionals not related to cleft care.
